# Mental Well-Being During Pandemic: The Role of Cognitive Biases and Emotion Regulation Strategies in Risk Perception and Affective Response to COVID-19

**DOI:** 10.3389/fpsyt.2020.589973

**Published:** 2020-11-05

**Authors:** Anna Schudy, Karolina Żurek, Marcelina Wiśniewska, Aleksandra Piejka, Łukasz Gawȩda, Łukasz Okruszek

**Affiliations:** ^1^Department of Cognitive Psychology and Neurocognition, Faculty of Psychology, University of Warsaw, Warsaw, Poland; ^2^Social Neuroscience Laboratory, Institute of Psychology, Polish Academy of Sciences, Warsaw, Poland; ^3^Experimental Psychopathology Laboratory, Institute of Psychology, Polish Academy of Sciences, Warsaw, Poland

**Keywords:** COVID-19, pandemic, mental well-being, emotion regulation, cognitive biases, risk perception

## Abstract

Both cognitive appraisals of risks associated with the specific disease and affective response to crisis situations have been shown to shape an individual response to pandemics. COVID-19 pandemic and measures introduced to contain it present an unparalleled challenge to mental well-being worldwide. Here, we examine the relationship between self-reported cognitive biases (CB) and emotion regulation skills (ER), COVID-19 risk perception and affective response, and mental well-being (MWB). Five Hundred and Eleven individuals completed General Health Questionnaire, Emotion Regulation Questionnaire, Davos Assessment of Cognitive Biases Scale (DACOBS) as well as scales measuring COVID-19 risk perception and affective response during the initial days of the epidemic in Poland. We used path and bootstrapping analyses to examine the hypothesis that CB may shape MWB during COVID-19 pandemic both directly and indirectly by (i) decreasing ER capacity and (ii) by increasing COVID-19 risk perception and affective response. Negative effect of CB and positive effect of ER via cognitive reappraisal on MWB were observed in participants. Furthermore, in line with our hypothesis, we observed indirect effects of CB via increased COVID-19 risk perception and affective response and decreased use of reappraisal strategy, which all, in turn, were related to MWB. Finally, we found an indirect effect of CB on MWB through double mediation of suppression strategies and COVID-19 affective response. Results of the current study suggest that CB, which have been shown to be linked to a variety of mental health symptoms in non-clinical populations, may exacerbate the impact of the COVID-19 pandemic on mental health outcomes.

## Introduction

The novel coronavirus outbreak in late 2019 in Wuhan (Hubei, China) and its rapid worldwide spread have led to the pandemic on a scale not seen since the Spanish flu epidemic in the early 20th century. The COVID-19 pandemic enforced abrupt changes in the functioning of world and state organs, healthcare systems, economy and education, as well as the everyday lives and habits of individual people. Up to March 2020 the virus started to spread across all the continents and at the time the epicenter of the pandemic was localized in Europe. Therefore, many countries, including Poland, implemented the preventive measures aiming at slowing down the COVID-19 spread and “flattening the curve” of infection increase by minimizing the number of concurrently active cases. Since the state of epidemic threat has been declared by the Polish government on March 13th (soon after the first fatal COVID-19 cases occurred), the most important strategies introduced in Poland were travel and gathering restrictions, mandatory quarantine, lockdown of educational institutes and an obligation of wearing masks while in public. Importantly, unlike some of the European countries with the highest infection rates (e.g., Italy and Spain), Poland did not implement any general lock-down.

During the previous viral outbreaks, such as the SARS epidemic in 2003, several factors that may shape the impact of the pandemics on mental well-being were established. Apart from demographic factors such as age, gender, education, employment status (1), some psychological variables were shown to be particularly important to mental well-being of individuals during epidemic. Lau et al. (1) showed that a sense of community connectedness was a major mitigating factor for stress associated with SARS outbreak. However, the recent COVID-19 outbreak, declared as a state of pandemic by the World Health Organization [(2), March 11th], caused countries to implement unprecedented preventive measures. The highly contagious nature of the COVID-19 viral agent led to introducing social distancing restrictions and lockdown type measures across countries. Thus, the buffering effect of the social environment might have weakened.

In search of psychological factors contributing to the individual response to the H1N1 outbreak, known as the swine flu, Rudisill (3) conducted a study using data of 944 British participants from a survey taken right before the start of the government's vaccination campaign. Results showed that higher H1N1 risk perception was a strong predictor of the intent to vaccinate oneself against the virus and undertaking avoidance behaviors. However, optimism about personal risk of contagion did not predict any of these actions. Similar results were obtained by Brewer et al. (4) in a meta-analysis showing that risk perception indeed drives actions (e.g., vaccination) and people tend to accurately perceive risk as lower after implementing protective strategies (5).

However, evidence accumulates on how easily the risk perception of a virulent agent can be biased [see review by (6)]. For example, Finucane et al. (7) showed that the more emotional impact a given risk has, the greater the risk itself seems. This phenomenon is known as the “affect heuristic.” Recent work found fear to be a crucial predictor of preventive behavior during the COVID-19 pandemic (8). While fear is known to increase the perception of risk, anger was found to diminish risk perception (9).

On the one hand risk perception may be influenced by exogenous factors e.g., media coverage, as shown by Chan et al. (10) on the case of Zika virus outbreak. On the other hand, the intrinsic human cognitive system is naturally biased, inter alia, in risk assessment (11). Previous research showed that both threatening context and a dispositional tendency to perceive infection risk as higher might be related to overperceiving disease cues (12). Thus, it is plausible that cognitive biases may affect risk assessment associated with COVID-19 pandemic. Yet, up to date, very few studies have addressed this issue [e.g., (13, 14)].

Cognitive biases are one of the core domains of social cognition. Biases (such as jumping to conclusions, attention to threat bias, belief inflexibility bias, external attribution bias) are studied extensively predominantly in neuropsychiatric populations, however they are also present in the general population to varying degrees [e.g., (15)]. Biased cognitions can influence affective states. In fact, cognitive biases and emotion regulation are intimately linked in various models of affective disorders [see review by (16)]. As cognitive biases may alter appraisal of an event they thus interfere with emotion regulation processes.

Effective emotion regulation is crucial for successful functioning in dynamic environments. The two most commonly used strategies of emotion regulation consist of re-evaluating a situation in order to diminish its emotional impact (cognitive reappraisal) and inhibiting outward expression of inner emotions (expressive suppression) (17). In a recent work of Restubog et al. (18) authors underline the important role that emotion regulation may play in maintaining psychological well-being during COVID-19 pandemic. In China, the drop in overall emotional well-being associated with the surge of COVID-19 reached 74% (19). A nationwide survey in which a total number of 14 000 respondents took part pointed out that the risk of contracting the virus, being in the high-risk group, relational issues and personal knowledge about the virus are some of the most important factors affecting mental well-being during pandemic.

Based on extensive literature research we identified several factors which may shape mental well-being during pandemic. It was shown before that cognitive biases are associated with psychopathology and may alter threat perception, while risk perception is greatly associated with emotional impact of the threat itself. At the same time, effective emotion regulation is associated with psychological well-being, also during pandemic. However, interactions of all aforementioned factors have not been investigated before. Thus, in the current study, we aimed at examining the relationship between self-reported cognitive biases and emotion regulation skills, COVID-19 risk perception and affective response, and its effect on mental well-being during pandemic. We hypothesized that cognitive biases, by interfering with emotion regulation processes, perception of risk and affective response to COVID-19, may decrease psychological well-being in time of pandemic.

## Methods

### Participants

Subjects were recruited for the study via online adverts 48 h after declaring the state of epidemic threat in Poland. We invited adults aged between 18 and 35 to complete an open online survey via Qualtrics. The data was collected during the period of 36 h. We collected the data of 511 individuals, yet after excluding subjects with outlying outcomes in variables of interest the final study sample amounted for 474 individuals. The sample in the current study was a convenience sample. Detailed demographic information of the final study sample is depicted in Table 1.

**Table 1:** Demographic characteristics of the study sample.

	**Female**	**Male**	**Other**	**Decline to answer**
Number of participants	378	90	4	2
Age in years [M(SD)]	23.01 (3.45)	23.75 (3.85)	24.0 (5.35)	24.5 (0.71)

The protocol of the study was accepted by the Ethics Committee at the Institute of Psychology, Polish Academy of Sciences. Prior to completing the online questionnaires participants were informed about the aim of the study and their right to withdraw at any moment. They were also insured about the anonymity of the data collected for the purpose of the study and that all analyses will be performed on the group level. Participants were not reimbursed for participation in the study.

### Materials and Procedure

Participants were asked to complete online surveys concerning their current general health problems, emotion regulation capacity, social-cognitive biases and COVID-19 risk perception, and affective response.

The online survey was prepared in accordance with the Checklist for Reporting Results of Internet E-Surveys (CHERRIES) (20). It consisted of 10 pages, each containing a number of items ranging from 7 to 40. Prior to launching the survey all online materials were previewed by five researchers from our team. In order to proceed to another page all questions had to be answered. Participants could not return to the previous page after they chose to go to the next one. Participation rate was 0.93, while the completion rate was 0.56. Only completed questionnaires were analyzed. We checked whether any IP Address appeared in the database more than once. Two IP Addresses duplicated but each of the entries contained a unique email address.

Mental well-being was assessed with the 30-item version of General Health Questionnaire [GHQ: (21)]. The Polish version of the GHQ-30 has excellent reliability (Cronbach's α = 0.97) and was shown to have a three-dimensional structure. Higher GHQ overall score signifies more psychopathological symptoms and, overall, lower mental well-being.

The capacity of emotion regulation was measured with Emotion Regulation Questionnaire [ERQ: (22); Polish version by Smieja and Kobylińska (23)^1^ ERQ is designed to assesses the two emotion regulation strategies: (1) cognitive reappraisal (CR)—reinterpretation of an emotional event in order to modify its meaning and change the emotional impact; (2) expressive suppression (ES)—attempt to hide and/or reduce experienced emotions (22). Smieja and Kobylińska (23) estimated Cronbach's alpha coefficients of each scale in Polish version between 0.74 and 0.85.

In self-assessment of social-cognitive biases we used 18-item version of Davos Assessment of Cognitive Biases Scale [DACOBS: (24), Polish 18-item version by Gawȩda et al. (25)]. The 18-item version of DACOBS includes four subscales: (1) subjective cognitive problems, (2) safety behaviors, (3) attributional biases, (4) social cognition problems. Authors of the Polish version reported a satisfactory level of reliability for the whole scale—Cronbach's alpha for the total scale of 0.84.

Additionally, we aimed at measuring COVID-19 risk perception and affective response to pandemic in our participants. We implemented a scale prepared for the purpose of the current study in which participants were asked to rate the perceived probability of various events related to COVID-19 pandemic (e.g., contact with virus carrier, developing severe symptoms etc.) and the level of worry these events may arise on seven-point Likert scale (1–“Definitely not”; 7–“Definitely yes”). While in the previous research we have analyzed specific factor subscales (26), the current study utilized overall risk perception (seven items, Cronbach's alpha = 0.80) and affective response (10 items, Cronbach's alpha = 0.70) scores, as each of the scales has shown adequate internal consistency.

### Statistical Analyses

The analyses were performed using SPSS version 26 and AMOS version 26.

We used path and bootstrapping analyses to examine the hypothesis that cognitive biases may shape mental well-being during COVID-19 pandemic both directly and indirectly by (i) decreasing emotion regulation capacity and (ii) by increasing COVID-19 risk perception and affective response. Sequential mediation was tested by entering cognitive biases, mental health symptoms, risk perception, affective response to COVID-19 pandemic to a path model in AMOS 26.

Model fit was assessed by using chi-square statistic to compare parameters of the model and the observed covariance matrix. Additionally, the goodness of fit was evaluated by using the comparative fit index (CFI) and root mean square error of approximation (RMSEA) (27). The significance of specific indirect pathways was examined by verifying whether 95% bootstrapped confidence intervals for any of the indirect effect contained the zero value.

## Results

Prior to examining the path model, we analyzed zero-order correlations between cognitive biases (DACOBS), mental health symptoms (GHQ), risk perception, affective response to COVID-19 (see Table 2).

**Table 2:** Zero-order correlations between cognitive biases, general health problems, risk perception and affective response to COVID-19.

	**(1)**	**(2)**	**(3)**	**(4)**	**(5)**	**(6)**
(1) DACOBS	1					
(2) GHQ	0.456^**^	1				
(3) ERQ cognitive reappraisal	−0.196^**^	−0.182^**^	1			
(4) ERQ expressive suppression	0.265^**^	0.116^*^	−0.005	1		
(5) COVID-19 risk perception	0.078	0.192^**^	0.074	−0.029	1	
(6) Affective response to pandemic	0.236^**^	0.300^**^	0.033	−0.102^*^	0.328^**^	1

** *p < 0.01 (two-tailed)*.

* *p < 0.05 (two-tailed)*.

We have included total score from DACOBS as a main independent variable and total GHQ-30 score as a main dependent variable. Furthermore, two types of emotion regulation strategies (cognitive reappraisal and expressive suppression) as well as COVID-19 specific items were included as mediators in the model. We investigated both direct effects of DACOBS, emotion regulation strategies and COVID-19 specific items on GHQ and indirect effect via mediator variables.

**Figure 1:**
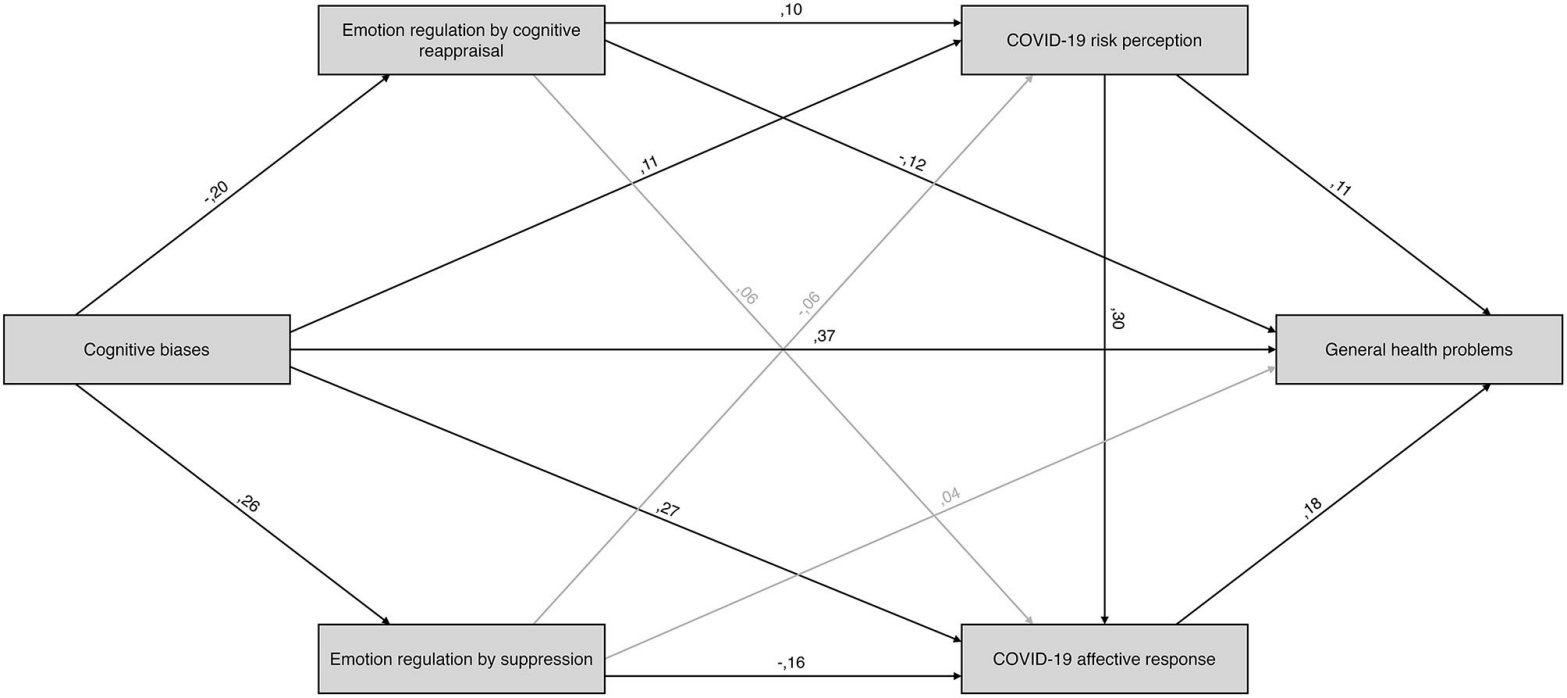
Path model of the associations between cognitive biases, general health problems, risk perception and affective response to COVID-19. Statistically insignificant paths are displayed in gray.

The final model, depicted in the Figure 1, had good fit to the data (χ^2^ (1) = 1.149, *p* = 0.284; RMSEA = 0.018, CFI = 0.999) and accounted for 27.2% of general health problems. We observed significant effects of cognitive biases (β = 0.368, *p* < 0.001), COVID-19 risk perception (β = 0.114, *p* < 0.01), affective response to pandemic (β = 0.184, *p* < 0.001) and emotion regulation via cognitive reappraisal (β = −0.124, *p* < 0.01) on general health problems.

More cognitive biases predicted higher COVID-19 risk perception (β = 0.112, *p* < 0.05), less emotion regulation via cognitive reappraisal (β = −0.196, *p* < 0.001) and more emotion regulation by suppression (β = 0.265, *p* < 0.001). We found a significant effect of emotion regulation via cognitive reappraisal (β = 0.096, *p* < 0.05) on COVID-19 risk perception. Additionally, significant effects of emotion regulation via suppression (β = −0.164, *p* < 0.001), COVID-19 risk perception (β = 0.297, *p* < 0.001), and cognitive biases (β = 0.269, *p* < 0.001) on COVID-19 affective response were observed.

Investigation of specific paths linking cognitive biases (independent variable) and general health problems (dependent variable) during pandemic revealed three significant indirect pathways. We found that the effect of cognitive biases on general well-being was positively mediated through use of emotion regulation strategy of cognitive reappraisal (β = 0.024, 95% CI = 0.008 to 0.05, *p* = 0.001), COVID-19 risk perception (β = 0.013, 95% CI = 0.002 to 0.033, *p* = 0.015) and affective response to pandemic (β = 0.049, 95% CI = 0.024 to 0.068, *p* < 0.001).

We also found that the effect of cognitive biases (independent variable) on affective response to COVID-19 (dependent variable) was mediated through emotion regulation via suppression (β = −0.043, 95% CI = −0.077 to −0.02, *p* = 0.001) and COVID-19 risk perception (β = 0.033, 95% CI = 0.004 to 0.068, *p* = 0.023).

Additionally, a double mediation explaining the effect of cognitive biases (independent variable) on affective response to COVID-19 (dependent variable) through emotion regulation by cognitive reappraisal and COVID-19 risk perception (β = −0.006, 95% CI = −0.015 to −0.001, *p* = 0.029) was found.

In total, the direct effect of cognitive biases on the outcome measure—general health problems—accounted for 81% of the total effect of DACOBS on GHQ variance (β = 0.456, *p* < 0.001), thus the impact of indirect effects on general health problems was very small (β = 0.087, *p* = 0.001).

## Discussion

In line with our initial hypothesis, we have found that cognitive biases may impact one's well-being in the wake of the COVID-19 crisis, both by directly affecting their perception and affective response to pandemics and by modulating effectiveness of one's emotion regulation strategies.

Firstly, we have observed that cognitive biases, as measured by self-assessment methods, may impact mental well-being via multiple possible pathways. The current study has observed a robust effect for the direct impact of cognitive biases on mental health problems in the general population during the COVID-19 crisis. While the relationship between cognitive biases and outcome measures was investigated so far mostly in clinical populations [e.g., patients with schizophrenia (28), patients with borderline personality disorder (29)], cognitive biases were also linked to the multiple detrimental outcomes, including psychopathological symptoms, e.g., psychotic-like experiences also in non-clinical populations (15). The current study provides additional support for the link between cognitive biases and overall mental health well-being, as indicated by a wide range of psychopathological outcomes measured by the GHQ. Moreover, the current study provides evidence that cognitive biases predict higher levels of disadaptive emotion regulation strategies (suppression) and lower levels of adaptive emotion regulation strategies (cognitive reappraisal) use in the general population. Furthermore, we observed an indirect effect linking cognitive biases with decreased mental-health well-being via reduced use of the cognitive reappraisal strategy. A similar effect was recently documented with regard to depressive symptoms—both disadaptive (brooding) and adaptive (positive reappraisal) strategies were observed to mediate the effects of cognitive biases on depressive symptoms in 119 healthy participants (30).

Secondly, higher levels of cognitive biases predicted higher COVID-19 risk perception in participants, which suggests that cognitive biases may be among factors shaping risk perception in response to pandemics crisis. Many recent studies have investigated the factors that underlie one's cognitive response toward COVID-19 pandemics, and identified both demographic [e.g., age: Gerhold (31)] and situational factors [e.g., being a health worker: Simione and Gnagnarella (32)], that predict COVID-19 risk perception. However, psychological factors including stress (32), dread (33) were also shown to be predictive on COVID-19 risk perception, thus emphasizing that subjective level of COVID-19 risk perception may be misshaped by numerous personal characteristics. The set of the specific cognitive biases assessed by the DACOBS is linked mostly to the social domain and include (i) increased interpersonal threat perception and avoidance, as well as (ii) subjective cognitive and social cognitive problems. Thus, the relationship between specific tendencies measured by the DACOBS and COVID-19 risk perception may be, at least partially explained, by the social nature of the COVID-19 transmission and focus on social distancing as a main preventive behavior during COVID-19 pandemics. Furthermore, the inconsistent nature of media coverage of the COVID-19 pandemics has been emphasized, e.g., during the first weeks of the pandemic, SARS-CoV-2 has been often compared, both by media and officials to the common flu which may have hindered the adequate risk assessment by individuals (Kumar, 03/27/2020), the effects of which may be particularly striking in individuals who report less-efficient cognitive capacities. Finally, it has been previously shown that the perceived threat from the A/H1N1is lower in the more self-efficacious individuals (34). As more self-efficacious individuals were shown to be more prone to produce subjective cognitive complaints both in clinical (35) and non-clinical (36) populations, further role of the cognitive biases and problem s as a mediator between self-efficacy and COVID-19 response should be investigated.

A robust effect of cognitive biases on COVID-19 affective response was also found in the current study. It has been previously shown, one's affective response to pandemic may be of more importance with regard to one's individual behavior compared to cognitive evaluations of risk associated with the virus (37). One of the most widely discussed cognitive biases in association with affective response is attention to threat bias. As Cisler and Koster (38) show in their review of models linking attention to threat bias and anxiety that this relation is likely to be moderated by emotion regulation strategies. In line with this claim we found that the effect of cognitive biases on affective response to COVID-19 pandemic was mediated by disadaptive emotion regulation strategy (expressive suppression), but for cognitive reappraisal strategy this mediation was not observed.

Only one of emotion regulation strategies assessed in the current study had a significant effect on COVID-19 risk perception. The effect of emotion regulation by cognitive reappraisal was positive—the tendency to use this strategy predicted higher COVID-19 risk perception.

Although it is commonly assumed that cognitive reappraisal is an adaptive emotion regulation strategy and expressive suppression is considered as one of the maladaptive ones, Gross (17) suggested that the emotion regulation strategy is only as adaptive as the context in which it is implemented is appropriate for its use. Bonanno et al. (39) found empirical evidence for the advantage of flexibility in emotion regulation strategies. In a longitudinal design study conducted after the 9/11 terrorist attacks on World Trade Center towers, authors found that flexibly implementing both emotion regulation strategies predicted lower levels of distress in college students after a year's time. In the case of COVID-19 pandemic Bonanno's et al. (39) findings seem especially relevant. The upsurging amount of contradictory information about the course of pandemic may require flexible implementation of various emotion regulation strategies to control psychological distress caused by COVID-19.

The observed direct effect of cognitive biases on general health problems explained the vast majority of GHQ variance in our study sample, while the effect of indirect effects linking those two variables was relatively small. As mentioned before, cognitive biases play an important role in numerous models of neuropsychiatric disorders (e.g., schizophrenia, social anxiety). Also, higher levels of cognitive biases are also found in at-risk populations [e.g., (40)]. Therefore, it may be hypothesized that the robust direct effect of cognitive biases on general health problems assessed in the current study in the general population may be linked to the general link between cognitive biases and psychopathology, rather than specific COVID-19 related circumstances.

Some limitations of the current study may be pointed out. Firstly, the cross-sectional design of the study does not provide insight into temporal relations between independent variables and GHP during pandemic. Secondly, cognitive biases are often considered as a mediator, rather than independent variable, e.g., it has been shown that cognitive biases mediate the relationship between childhood trauma and psychotic-like experiences (41), especially in research involving clinical or subclinical neuropsychiatric populations. Here, we did not assess any variables which may underlie prevalence for expressing more cognitive biases, thus the current observations may not fully account for potential independent variables e.g., childhood trauma [e.g., (41)], social adversities [e.g., (42)] etc. Lastly, a number of other factors such as history of mental illness, genetic vulnerability, social adversities, psychological individual differences or actual contact with the COVID-19 disease may also affect mental well-being during pandemic. Future research should address this limitation by including variables likely to impact cognitive biases as well as general health problems into the model.

## Data Availability Statement

The raw data supporting the conclusions of this article will be made available by the authors, without undue reservation.

## Ethics Statement

The studies involving human participants were reviewed and approved by Ethics Committee at the Institute of Psychology, Polish Academy of Sciences. The patients/participants provided their written informed consent to participate in this study.

## Author Contributions

ŁO, AP, MW, and KŻ contributed to conception and design of the study. ŁO and AS performed the statistical analysis. AS and ŁO wrote the first draft of the manuscript. KŻ and AP wrote sections of the manuscript. MW prepared data visualizations. All authors contributed to manuscript revision, read, and approved the submitted version.

## Conflict of Interest

The authors declare that the research was conducted in the absence of any commercial or financial relationships that could be construed as a potential conflict of interest.

## Footnotes

^1^https://spl.stanford.edu/sites/default/files/polish.pdf
