# Supplementary material for: Mental Well-Being During Pandemic: The Role of Cognitive Biases and Emotion Regulation Strategies in Risk Perception and Affective Response to COVID-19
Source: Front Psychiatry. 2020 Nov 5;11:589973. doi: 10.3389/fpsyt.2020.589973 (PMC7678487; doi:10.3389/fpsyt.2020.589973)
Supplement: Supplementary file 1 [file Data_Sheet_1.PDF]

**Checklist for Reporting Results of Internet E-Surveys (CHERRIES)**

| ***Checklist Item*** | ***Explanation*** | ***Page Number*** |
|---|---|---|
| Describe survey design | We invited adults aged between 18 and 35 to complete an online survey via Qualtrics. The sample in the current study was a convenience sample. | 3 |
| IRB approval | The protocol of the study was accepted by the Ethics Committee at the Institute of Psychology, Polish Academy of Sciences. | 3 |
| Informed consent | Prior to completing the online questionnaires participants were informed about the aim of the study and their right to withdraw at any moment. They were also insured about the anonymity of the data collected for the purpose of the study and that all analyses will be performed on the group level.” | 3 |
| Data protection | The data collected in the project were fully anonymous and analyzed on the group level. | 3 |
| Development and testing | The survey was previewed by five researchers from our team. | 4 |
| Open survey versus closed survey | Open survey | 3 |
| Contact mode | Internet | 3 |
| Advertising the survey | The survey was distributed on Facebook groups, mostly devoted to student communities from different Polish universities and faculties. | 3 |
| Web/E-mail | a web survey | 3 |
| Context | The survey was distributed on Facebook groups, mostly devoted to student communities from different Polish universities and faculties. | 3 |
| Mandatory/voluntary | Voluntary | 3 |
| Incentives | No incentives were offered to the volunteers. | 3 |
| Time/Date | “Subjects were recruited for the study via online adverts 48h after declaring the state of epidemic threat in Poland. (...) The data was collected during the period of 36 hours.” | 3 |

| | | |
|---|---|---|
| Randomization of items or questionnaires | NA | |
| Adaptive questioning | NA | |
| Number of Items | The number of items per page ranged from 7 to 40. | 4 |
| Number of screens (pages) | 10 | 4 |
| Completeness check | Yes, all questions had to be answered in order to submit the results. | 4 |
| Review step | Participants could not return to the previous page after they chose to go to the next one. | 4 |
| Unique site visitor | NA | |
| View rate (Ratio of unique survey visitors/unique site visitors) | NA | |
| Participation rate (Ratio of unique visitors who agreed to participate/unique first survey page visitors) | 916 (agreed to participate) / 989 (visited the first page/informed consent) = 0.93 | 4 |
| Completion rate (Ratio of users who finished the survey/users who agreed to participate) | 511 / 916 = 0.56 | 4 |
| Cookies used | NA | |
| IP check | We checked whether any IP Address appeared in the database more than once. Two IP Addresses duplicated but each of the entries contained a unique email address. | 4 |

| | | |
|---|---|---|
| | | |
| Log file analysis | NA | |
| Registration | NA | |
| Handling of incomplete questionnaires | Only completed questionnaires were analyzed. | 4 |
| Questionnaires submitted with an atypical timestamp | NA | |
| Statistical correction | NA | |

This checklist has been modified from Eysenbach G. Improving the quality of Web surveys: the Checklist for Reporting Results of Internet E-Surveys (CHERRIES). J Med Internet Res. 2004 Sep 29;6(3):e34 [erratum in J Med Internet Res. 2012; 14(1): e8.]. Article available at https://www.jmir.org/2004/3/e34/; erratum available https://www.jmir.org/2012/1/e8/. Copyright ©Gunther Eysenbach. Originally published in the Journal of Medical Internet Research, 29.9.2004 and 04.01.2012.

This is an open-access article distributed under the terms of the Creative Commons Attribution License (https://creativecommons.org/licenses/by/2.0/), which permits unrestricted use, distribution, and reproduction in any medium, provided the original work, first published in the Journal of Medical Internet Research, is properly cited.
